# A deep-learning framework for spray pattern segmentation and estimation in agricultural spraying systems

**DOI:** 10.1038/s41598-023-34320-7

**Published:** 2023-05-09

**Authors:** Praneel Acharya, Travis Burgers, Kim-Doang Nguyen

**Affiliations:** 1 Department of Mechanical Engineering, South Dakota State University, Brookings, SD 56007 USA; 2 Raven Industries, Inc., Sioux Falls, SD 57104 USA; 3 Department of Mechanical and Civil Engineering, Florida Institute of Technology, Melbourne, FL 32901 USA

**Keywords:** Mechanical engineering, Computational science, Scientific data, Information technology

## Abstract

This work focuses on leveraging deep learning for agricultural applications, especially for spray pattern segmentation and spray cone angle estimation. These two characteristics are important to understanding the sprayer system such as nozzles used in agriculture. The core of this work includes three deep-learning convolution-based models. These models are trained and their performances are compared. After the best model is selected based on its performance, it is used for spray region segmentation and spray cone angle estimation. The output from the selected model provides a binary image representing the spray region. This binary image is further processed using image processing to estimate the spray cone angle. The validation process is designed to compare results obtained from this work with manual measurements.

## Introduction

Crop spraying is one of the most fundamental processes in agriculture carried out to apply herbicides, pesticides, fertilizers, and other necessary chemicals to crops. A key problem with some current crop spraying systems is that only a small percent of the spray sticks to the crop as intended^1^. Due to plants’ hydrophobic properties and spray patterns, a large portion of sprayed chemicals bounce off plants, land on the ground, and become part of the chemical runoff that flows to streams and rivers, often causing serious pollution^2^. Researchers discovered that pesticides were detected in $90\%$ of agricultural streams, $50\%$ of wells and $33\%$ of deep aquifers sampled across the U.S.^3^. Studies^4–6^, related this chemical runoff problem to the pattern of spray emitted from nozzles. Agricultural nozzles’ effectiveness is determined by many different factors, among which the most important are droplet size and velocity and *spray pattern*. In earlier work^7^, we developed a tool for measuring droplet size and velocity. Thus, there is a critical need for precise measurements of *spray pattern* to study the efficacy of sprayer systems in the retention of agricultural spray, and adjust the spray pattern to achieve optimal retention, and hence minimize the chemical runoff that pollutes the soil-water-plant systems.

### Motivation and scope

This paper focuses on extracting two critical characteristics of an agricultural sprayer system given an input image: segmentation of a spray region and estimation of spray cone angle. Successful segmentation to determine the spray region in a given image will provide useful information about spray patterns. When spray patterns do not perform as expected it can lead to over or under-spraying of chemicals causing unnecessary waste of chemicals. Therefore, measuring spray patterns is essential in understanding the overall effectiveness of sprays. Similarly, spray cone angle is crucial to estimating the reach of a spray. In theory, an end-user can choose a nozzle system that satisfies the desired characteristics of a spray in accordance with the system specifications. For example, an 8005 flat-fan nozzle is supposed to generate a spray cone angle of 80 degrees when operating at a rated pressure of 40 psi with a discharge rate of 0.5 gallons per minute. Specialized instruments such as patternators can be used in a lab environment to understand and study spray patterns. For example, when investigating the performance of different hand-held sprayer types, researchers manually measured the spray angle while following the ISO 5682-1 standard using a dedicated testbed^8^. However, these specifications are theoretical values and they may change drastically when environmental factors come into play. Even a sprayer system rated for 80 degree spray cone angle can produce different actual angle values as the actual system pressure varies along the flow.

Spray angles are also influenced by dimensions, liquid characteristics, and density of the ambient to which the liquid was sprayed^9^. Yu et al. tested 18 different nozzles and noticed a spray angle difference of up to $10\%$ as compared to the manufacture-rated nozzle spray angle^10^. More importantly, the techniques developed in this paper will facilitate automated recognition and measurement of spray patterns and angles in a given spray image. This method will be much more economical than manual measurements of spray patterns by a human operator. The approach discussed in this work can be employed as a feedback mechanism to control and adjust the spray patterns in the field to achieve more desirable spray efficacy. In addition, this research lays a foundation that can be integrated into the early phases of nozzle design and investigation in lab settings.

The scope of this work is to develop an advanced AI-enable tool to recognize, measure, and quantify spray patterns from agricultural nozzles. To achieve this goal, the research emphasizes the importance of developing a machine-learning model that is light on the number of parameters while maintaining the desired performance. A model with fewer parameters will generate output faster and require less memory resources. In addition, this work also clearly illustrates a pathway to leverage deep learning and computer vision to estimate spray boundary and spray cone angle.

A natural continuation of this work is to develop a classification neural network that takes the measurement generated by this research and outputs the quality of the spray of interest. This neural network will be trained and supervised with ground-truth data labeled by a human expert. The AI agent will also take into account the type of crop as well since a spray good for one crop may be poor for another crop because the hydrophobic properties of different plants may vary. Another future direction would be to expand on this research to investigate the effects of other factors like wind, drift, tractor motion, or rough terrains on the spray region and cone angle.

Deep learning plays a central role in this work. This is driven by the fact that deep-learning methods have recently shown impressive results in computer vision and machine learning. For example, deep learning has been implemented for successful classification^11–13^, detection^7,14–16^, segmentation^17,18^, and reinforcement learning^19–21^ to name a few. Furthermore, this work focuses on using images taken from a common phone camera instead of an expensive high-speed camera which makes it more accessible. In summary, the motivation of this research is to leverage the potential of deep learning and computer vision to address one of the most fundamental problems in precision agriculture, namely measuring agricultural spray characteristics.

### Related work in estimation of spray cone angle

The majority of the existing work on estimating spray cone angle focused primarily on fuel spray systems and very little work on agricultural applications. In particular, artificial light has been added to generate high contrast images to help with the detection of spray boundary which is crucial for spray cone angle computation^22^. Similarly, a black background was added in addition to the use of para flashes for manipulating the lighting condition^23^. A special charged-coupled device (CCD) camera is used to collect images^24^. Existing computer vision software is used to manually measure spray cone angle^25^. A CCD camera with a stroboscope was used to capture the image which is processed to estimate the spray angle^26^. To better understand the performance of liquid rocket engines with pintle injectors, spray angles is manually measured of the time-averaged spray images in addition to using a CCD camera^27^. Similar use of a high-speed CCD camera is used for spray angle estimation^28^.

These existing studies used specialized cameras to capture images whereas, in our project, we work with images recorded using a common cellphone camera. More importantly, the effectiveness of these existing techniques relies on the environmental lighting condition. Setting up and tuning the lighting condition is time-consuming and requires multiple trial and error iterations to get to the desired lighting condition. To avoid this issue, we designed a deep neural network to transform images obtained from the camera into a binary image to extract the spray cone angle. This binary image has only two distinct regions: the white region represents spray while everything else is black and represents the background. Therefore, our proposed method minimizes the dependence on the lighting conditions. This saves a lot of time and makes the system accessible to the average user.

### Related work in deep learning for image segmentation

Determining the spray patterns in an image can be achieved through segmentation. As with many branches of computer vision in this decade, a lot of research has been carried out focusing on image segmentation. Since we are dealing with input images, a convolution-based segmentation is a natural choice. Seminal work^29^ is regarded as one of the early segmentation techniques that employ a convolution-based approach. Though it is a revolutionary model at the time, many new improved models have been introduced to address some of the shortcomings of this work. For example, ParseNet was developed^30^ to utilize the global context of the scene which leads to a reduction in the loss of the scene-level semantic context. Another widely recognized and used model for segmentation is U-Net^31^. U-Net enables precise segmentation using fewer training images^29^. In addition, with a higher number of feature channels, U-Net allows the network to propagate context information to higher resolution layers.

There have been many variations of U-Net to target different applications. For example, recent work^32^ implemented a U-Net-inspired model for sea bottom detection based on sonar data. A light model of U-Net, called Mobile-Unet, was developed for fabric defect detection^33^. In addition, a variation called S3D-Unet is proposed for brain tumor segmentation^34^. The segmentation model in this paper is also inspired by U-Net. The motivation for choosing the U-Net model, beside being widely successful for different applications, is that it was designed to deal with a limited data set while achieving desirable performance.

**Figure 1:**
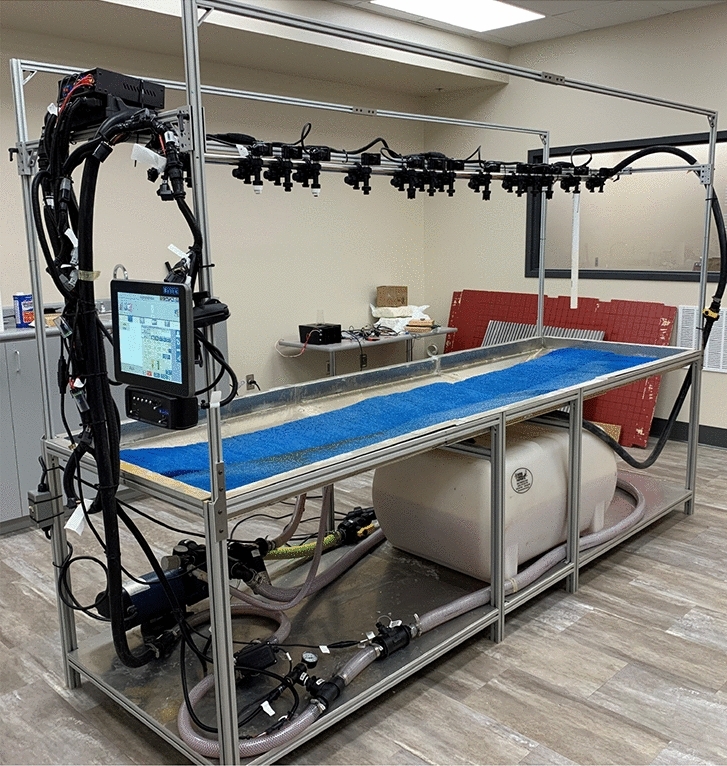
Experimental set-up with crop spray nozzles for data collection.

Two different models for spray pattern segmentation were developed in this paper. Both will be trained with experimental data collected with our spray system test bed shown in Fig. 1. In particular, the test bed includes the Raven Industries Hawkeye2 pulse width modulation (PWM) system, which operates at 10Hz while maintaining constant boom pressure. PWM spray system is the most advanced technology to obtain variable rate spray application without varying the operative sprayer parameters^35^. In addition, the user interface is used to control flow rate and different spray nozzles to generate different spray patterns. A comparative study will be presented to compare the performance of these new models as well as of the original U-Net model. The key contributions of this work are as follows:

- Two new deep-learning models for spray pattern segmentation.
- Training, validation, and comparison of the two deep-learning models for spray region segmentation with the U-Net model.
- Showing that $1\times 1$ convolution can be prominently used in deep learning-based segmentation models through the training results of the two proposed models.
- New techniques for estimating the spray cone angle based upon deep-learning model outputs for agricultural applications.
- Experimental results of spray cone angle determination using the two new models for images taken with a common phone camera. In addition, the performance of the proposed models is not affected by environmental factors such as lighting and contrast conditions.

## Data processing operations

**Figure 2:**
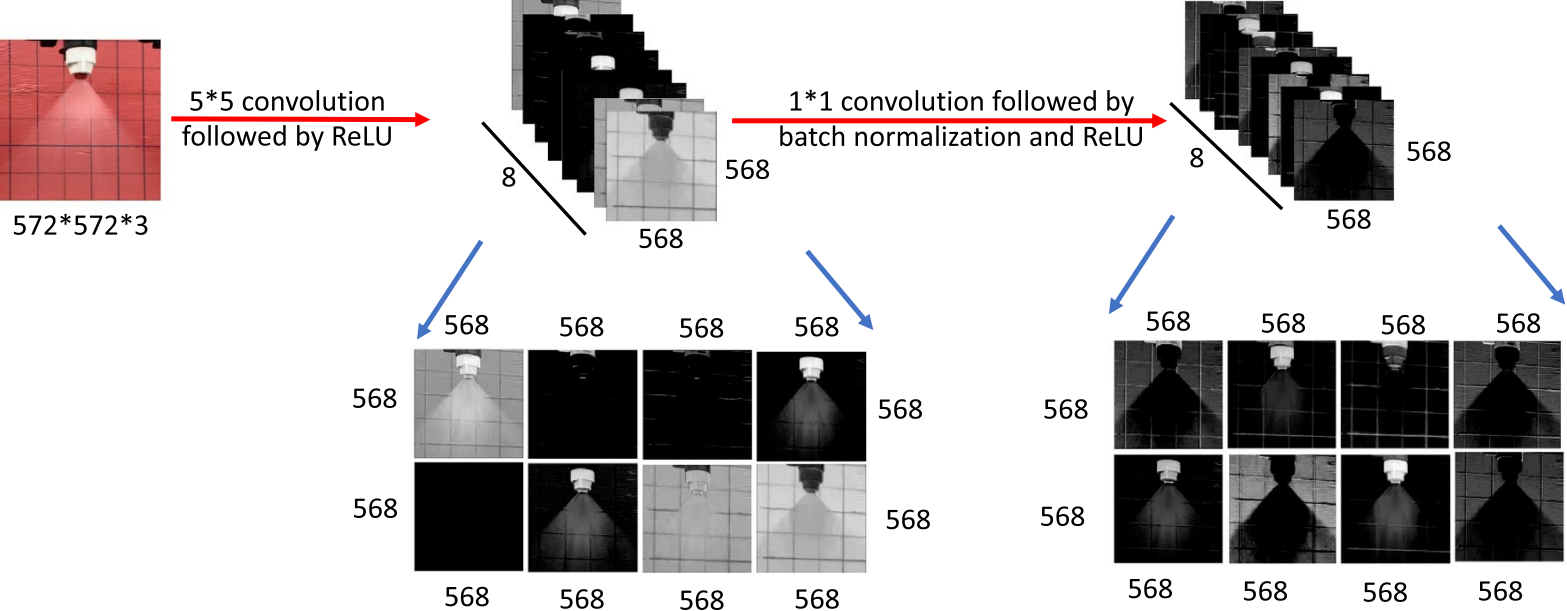
An input image going through multiple operations: Eight $5\times 5$ convolution filters are applied to the input RGB image resulting in an output of $568\times 568\times 8$. Since ReLU and batch normalization preserve the size, the size obtained by applying convolution will remain the same even after applying these operations. Convolution extracts important features from the image whereas ReLU is an activation function that restricts the output of the convolution operation in a certain range.

**Figure 3:**
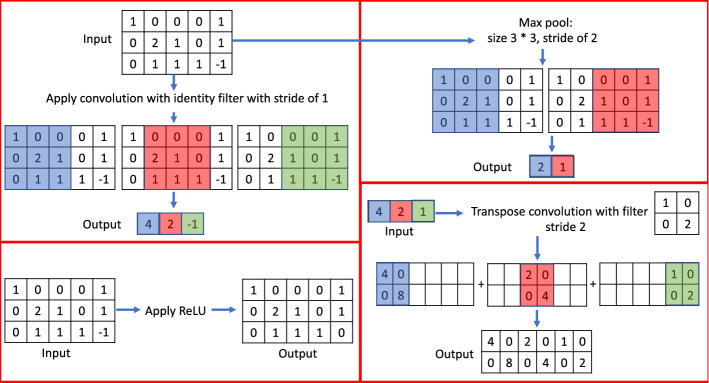
Illustration of applying basic operations including convolution, max pooling, transpose convolution, and ReLU activation to different inputs.

Given the objectives described in the last section, a fundamental process is to take an input image of a spray, process the data, and then extract critical features that define a spray pattern to be detected. Figure 2 illustrates the building-block operations of this fundamental process. The operations are described as follows:

- A $5\times 5$ convolution followed by ReLU activation is applied to an input image of size $572\times 572\times 3$. The resultant outputs are 8 binary images, each of size $568\times 568$, stacked one after another resulting in a total data size of $568\times 568\times 8$. More in-depth discussions about the operation are elaborated below:
  - **Convolution** is the process of applying a filter to an input image. A $5\times 5$ convolution means a filter of size $5\times 5$ is used for a given input. If the stride is one, the filter will move one pixel in each step until the entire input is processed. For example, the top left panel in Fig. 3 shows the result of applying an identity filter with a stride of 1 to a given input. The result of applying convolution in Fig. 3 is obtained by the following steps: First, the $3\times 3$ identity filter is placed over the image patch of the same size. Second, element-wise multiplication is done with the entry of the image patch and the identity filter, and the entries of the resulting matrix are summed. Mathematically, it can be written as, $1*1 + 0*0 + 0*0 + 0*0 + 2*1 + 1*0 + 0*0 + 1*0 + 1*1 = 4$. Since the stride is 1, in the next step, the identity filter moves one pixel and repeats the calculations until the entire image is processed. As a result for the next patch the output is $0*1 + 0*0 + 0*0 + 2*0 + 1*1 + 0*0 + 1*0 + 1*0 + 1*1 = 2$. For the last patch, the output is $0*1 + 0*0 + 1*0 + 1*0 + 0*1 + 1*0 + 1*0 + 1*0 - 1*1 = -1$. Thus, the result of applying convolution with identity filter is $[4, 2, -1]$ as depicted in Fig. 3. In Fig. 2, since we use 8 different filters, the process results in 8 outputs. The values in the filters’ entries are trained by a training process discussed in the next section.
  - **Activation** is the process of applying a function to each entry of the given input. The Rectified Linear Unit (ReLU) is one of such activation functions employed in this work. Since ReLU is defined as $ReLU(x) = max(0, x)$, negative values are converted to value zero. For example, the bottom left panel in Fig. 3 shows the result of applying ReLU activation to the input. In addition to ReLU, we also use sigmoid ($\textrm{Sigmoid} (x) = \frac{1}{1 + e^{-x}}$) as an activation function. No matter which activation function is used, the process of applying activation to the input remains the same. Activation functions are essential in deep learning, it is common to see activation being applied to the output from operations such as convolution and transpose convolutions. It is also worth noting that applying a filter does not change the size of the data.
  - **Transpose convolution** is the process of upsampling a given input by applying a filter. A $2\times 2$ transpose convolution means the filter size of $2\times 2$ is used. For example, the bottom right panel of Fig. 3 shows the result of applying a transpose convolution with a filter of size $2\times 2$ with a stride of 2 to the given input. In Fig. 3, for transpose convolution, the first entry of the input is 4 which is multiplied by the given filter. Similarly, the second and third entries of the input are multiplied by the filter. In this particular example, given a stride of 2, each intermediate output is stacked to get the final output as seen in Fig. 3. With a different stride size, more steps might be required to get the result again.
- A $1\times 1$ convolution followed by batch normalization and ReLU activation is then applied to the output of size $568\times 568\times 8$ coming out from the last process. Batch normalization is a technique of normalizing an input. Similar to applying an activation function, batch normalization does not alter the size of the input data. Batch normalization is a relatively new idea in the field of deep learning. Batch normalization helps in the training of deep neural networks^36^. In this work, batch normalization is carried out to the output of $1\times 1$ convolution. Since batch normalization preserves size, the output size from batch normalization is still $568\times 568\times 8$. Finally, ReLU activation is applied to the output of batch normalization. As the activation function also preserves size, the output size again remains $568\times 568\times 8$.
- The output of these operations contains feature maps of the same size and preserves all the key characteristics that define a spray pattern. These feature maps will then be processed for spray pattern segmentation.

## Neural network architecture

For spray pattern segmentation, we developed two deep-learning models, namely *Model A* and *Model B*, whose layer architectures are listed in Table 1 with the input size, output size, and all the operations carried out in each layer. A layer is a collection of operations. For example, layer 1 (down-sampling) takes an input image of size ($572\times 572\times 3$) and outputs a result of size ($568\times 568\times 8$). Operations carried in layer 1 (down-sampling) can be written in compact form as follows: input size ($572\times 572\times 3$), convolution ($5\times 5$, stride 1), activation (ReLU), convolution ($1\times 1$, stride 1), batch normalization, activation (ReLU), output size ($568\times 568\times 8$). Down-sampling and up-sampling are two different sets of operations elaborated as follows:

**Down-sampling**

- $2\times 2$ max pooling operation with stride of 2.
- $5\times 5$ convolution with stride of 1 followed by ReLU activation.
- $1\times 1$ convolution with stride of 1 followed by batch normalization and the resultant feature map passed through ReLU activation.

**Up-sampling**

- $2\times 2$ transpose convolution with stride of 2 is followed by batch normalization and the resulting feature map pass through ReLU activation.
- Stacking of feature maps.
- $1\times 1$ convolution with stride of 1, padding of one pixel throughout the border is followed by ReLU activation.

Essential to the down-sampling process is the **max pooling** operation. Max pooling calculates the maximum value for each patch in the given input resulting in the suppression of values except the maximum. As with convolution, the patch size has to be defined. For example, $3\times 3$ max pooling would mean a patch size of $3\times 3$. The top right panel in Fig. 3 shows the result of applying max pooling with a size of $3\times 3$ and a stride of 2 to the given input. The result of the max-pooling operation can be obtained by finding the maximum value in each patch: in the first patch, the maximum value is 2 and in the second patch, the maximum value is 1.

In both proposed models (refer to Table 1 for in-depth steps), convolution plays a vital role in the neural network architecture. The goal of convolution operations in the case of down-sampling is to gradually increase the depth of the feature map. In contrast, for up-sampling, the depth of the feature map gradually decreases with the help of transpose convolutions. One major operation that links the output from the down-sampling layer to the up-sampling layer is the stacking (concatenate) operation. The result obtained after the max-pooling operation (down-sampling) for any layer is stacked with the result obtained from transpose convolution for the same layer (up-sampling) as seen in Table 1. This operation in Table 1 is known as a stack. For example, the output feature map after max-pooling operation in layer 2 (down-sampling) is stacked with the output feature map after transpose convolution in layer 2 (up-sampling) to generate an output of size $284\times 284\times 16$. In the case of layer 6, the output feature map after the max-pooling operation is stacked with the output feature map after the last $1\times 1$ convolution operation of the same layer to output a feature map of size $14\times 14\times 256$ in layer 5 (up-sampling).

**Table 1:**
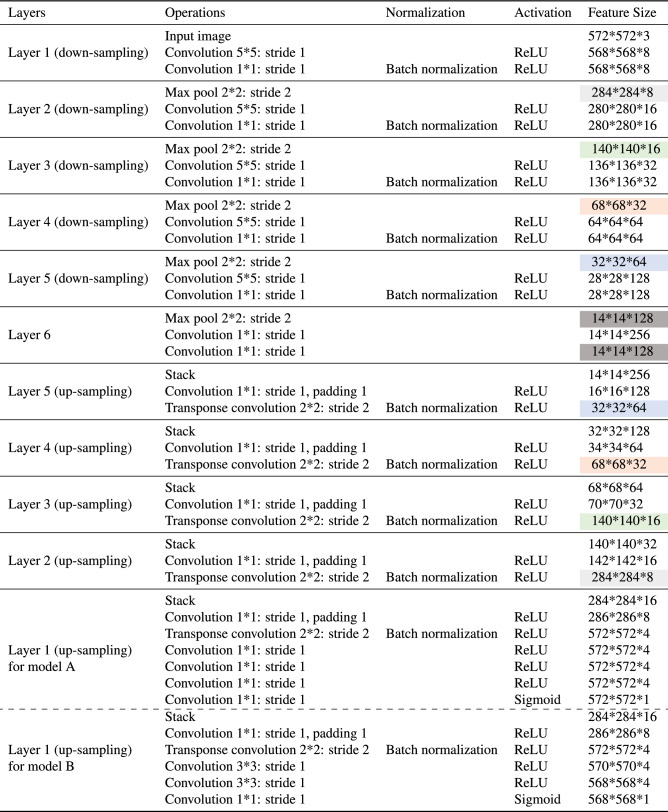
Network architecture for both models: *Model A* and *Model B*. Similar color regions in feature size column indicates the features that get stacked together.

In summary (refer to Table 1 for in-depth steps), if we repeat above down-sampling operations (two convolutions and max-pooling) five times with an input image of size $572\times 572\times 3$ pixels, we obtain feature map size of $14\times 14\times 128$. This output (obtained in layer 6) is then stacked with the result obtained by applying two convolutions. The resulting feature map of size $14\times 14\times 256$ is passed through the one convolution followed by a transpose convolution. The operation (stacking followed by a convolution and a transpose convolution) is also repeated five times resulting in the feature of size $572\times 572\times 4$. In the case of *model A*, four $1\times 1$ convolutions are applied at the end to obtain the output image of size $572\times 572\times 1$. In the case of *model B*, two $3\times 3$ convolutions are applied at the end to obtain the output image of size $568\times 568\times 1$. Both models take in the input image and output the binary mask for segmentation.

### Difference among models in terms of network architecture

The two new segmentation models are distinct as compared to the well-established U-Net model^31^. A significant difference from U-Net is the use of $1\times 1$ convolution in both *Model A* and *Model B*. This allowed us to significantly reduce the total number of trainable parameters in both proposed models.

As seen in the Table 2, U-Net has around 69 times more parameters compared to both *Model A* and *Model B*. According to work in^37^, SegNet, which is another popular segmentation model, has around 30 million parameters. A similar observation can be made in terms of model total size as shown in Table 2. Both of our proposed models are also a layer deeper as compared to U-Net. In general, a smaller number of parameters should take less time to complete a segmentation task for the same input image compared to a model with a larger number of parameters. Thus, there is a significant advantage of having a model with a fewer number of parameters. In addition to the reduced number of parameters, emerging techniques such as batch normalization have been introduced to both *Model A* and *Model B*. At this stage, in terms of model architecture, both proposed models have a significant advantage over U-Net for spray pattern recognition. The next sections will elaborate on the training and compare their performance.

**Table 2:** Comparison of three models in terms of network architecture.

Model	Trainable parameters	Estimated total size (MB)
*Model A*	449,317	195.44
*Model B*	449,553	185.21
U-Net Model	31,031,745	1146.01

## Training

We organize all date into three distinct groups: training, validation, and test. Images contained within the **training** dataset provide the AI agents with a large set of labeled examples that they can use to learn how to recognize spray patterns. The deep-learning models are trained by being presented with many examples of input data along with the corresponding correct output for each input. By analyzing and processing these examples, the algorithm can adjust its parameters to minimize the difference between its predicted output and the actual output via an optimization scheme. The **validation** dataset enables us to gain insight into the overall performance of our models when processing images that have not been previously used for training. While our AI models compute the loss of the validation dataset on each epoch, they do not learn from this data. The parameters of the deep-learning models remain fixed during validation loss computation, evaluating the model performance on data the models have not been trained on. The purpose of the **test** dataset is to evaluate model performance on images that the AI models have never encountered before. In addition, it represents real-world scenarios where different spray nozzles with varying lighting conditions and experimental settings produce a variety of spray patterns.

The image augmentation pipeline was used to increase the number of images and reduce data over-fitting. The image augmentation pipeline involved using one of the following operations: resizing the original image to the height and width of 572, central crop with a window size of $572\times 572$, and resizing to the size of $572\times 572$ with a horizontal flip. Out of three augmentation operations, one of them is randomly selected to both input and the desired output. To generate the desired output, the region around the spray is drawn for each image in the database which is used to generate a binary mask. A mask is a one-channel image with white pixels representing spray and black pixels representing the background.

At this stage, all input images and corresponding masks are of size $572\times 572$. Using these input images and masks, a database is built containing 1107 images. Each model takes an input size of $572 \times 572$ and outputs a single channel image of different sizes as seen in Table 1. Based on the model, the mask is resized again if necessary to fit the model’s desired size. For example, *Model B* output image has a height and width of 568. Whereas, the *U-Net* output image has a height and width of 388. In addition, the output from all models has a value between 0 and 1 because of the sigmoid function in the last layer. As a result, the ground truth is also scaled to the value of 0 to 1 before computing the loss function. Binary cross-entropy is used as the loss function and is defined below:

1 $$\begin{aligned} L_x = - \frac{1}{W \times H} \sum _{i=1}^{W} \sum _{j=1}^{H} \quad \bigg \{ y(i,j) \times log[{\hat{y}}(i,j)] + \quad (1-y(i,j)) \times log[ 1- {\hat{y}}(i,j)] \bigg \} \end{aligned}$$

where $L_x$ is the loss value for a given input image *x*, *y* is the desired output image, ${\hat{y}}$ is the generated image (output) by the model, *y*(*i*, *j*) and ${\hat{y}}(i,j)$ are the pixel values at $i^{th}$ row and $j^{th}$ column, and *H* and *W* are the output image’s height and width. The lowest bound for the log function in equation (1) is set to $-100$ to avoid singularity. With the loss function defined, the stochastic gradient descent (SGD) algorithm with momentum is used to minimize the loss function value during the training process.

To train the model, the database is divided into two groups: $80\%$ of images and their masks are used for training purposes and the remaining $20\%$ are used for validation. Let $\Omega _T$ be the set containing images used for training the model and $\Omega _V$ be the set containing images used for validation. The training loss is defined as $L_T = \frac{1}{n(\Omega _T)}\sum L_x,~ \forall x\in \Omega _T$, and the validation loss is defined as $L_V = \frac{1}{n(\Omega _V)}\sum L_x,~ \forall x\in \Omega _V$, with $n(\Omega _T)$ and $n(\Omega _V)$ representing the total numbers of elements in the given sets.

The hyperparameter values used for the training process are reported in Table 3. Though this work does not explore systematic ways to achieve optimal values for hyperparameters, currently selected values are not random either. The key driving factors for selecting values listed in Table 3 are as follows:

1. Higher values of batch size were not used to preserve memory while lower values of batch size lead to a lot of oscillations for training loss.
2. Larger learning rates lead to high oscillations in the validation loss while lower values were not explored as the model would take more time to converge.
3. Weight decay is used as a way to introduce regularization in the model, making the model more robust.

**Figure 4:**
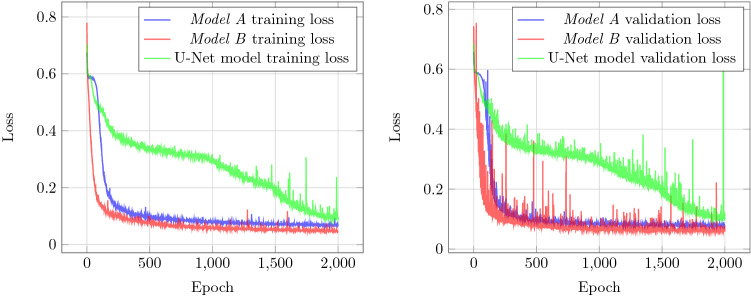
Left: Training loss for all three models. Right: Validation loss for all three models.

With the above-selected values, all three models (*model A*, *model B*, U-Net) are trained using the Pytorch framework. During the training process, the training loss $L_T$ and validation loss $L_V$ are computed at every epoch and can be seen in Fig. 4.

### Difference between models in terms of training time and performance

For the training, we employed the high-performance computing facility with an Intel Xeon Gold 6148 (2.4 GHz, 14nm) CPU and an NVIDIA Tesla P100 GPU. All three models were trained using the database discussed above and hyperparameters in Table 3. The training hardware is also kept constant and the training progress made by the models during this time can be seen in Table 4. The results presented in Table 4 show that the U-Net model takes approximately 40 hours more to train as compared to *Model A* and *Model B*. In addition, the U-Net model does not perform better when compared to both *Model A* and *Model B* in terms of training loss and the validation loss as indicated by Fig. 4. Beside binary cross-entropy loss, model performance is measured using additional metrics. To do so, a threshold is applied to the output of the model where the pixel value greater than 0.5 is replaced with 1 and 0 everywhere else. The following metrics for each ground truth in the validation set are computed:

- True Positive (TP) is the number of pixels that the model correctly predicts the pixel value of 1 when compared to ground truth.
- True Negative (TN) is the number of pixels that the model correctly predicts the value of 0 when compared to ground truth.
- False Positive (FP) is the number of pixels that the model predicts the value of 1 but the ground truth value for the pixel is 0.
- False Negative (FN) is the number of pixels that the model predicts the value of 0 but the ground truth value for the pixel is 1.

with TP, TN, FP, FN defined, commonly used segmentation metrics can further be computed: mean accuracy (MA), mean dice coefficient (MDC), mean intersection over union (MIoU).

**Table 3:** Training parameters.

Parameters	Values
Batch size	16
Total epoch	2000
Learning rate	0.001
Momentum	0.9
Weight decay	0.0001

**Table 4:** Training performance for all three models. .

Model	Total epochs	Training time	Average epoch in 1 h
Model A	2000	12.359 hours	161.825
Model B	2000	12.707 hours	157.393
U-Net model	2000	52.17 hours	38.336

Mean Accuracy (MA) computes the mean pixel accuracy between ground truth and output from the model and is defined as:

2 $$\begin{aligned} MA = \frac{1}{N} \sum _{i=1}^{N} \frac{TP + TN}{TP + TN + FP + FN}, \quad \text {N is the total number of images.} \end{aligned}$$

Mean Dice Coefficient (MDC) computes the similarity between the model-generated output image and the ground truth and is defined as:

3 $$\begin{aligned} MDC = \frac{1}{N} \sum _{i=1}^{N} \frac{2*TP}{2 * TP + FP + FN}, \quad \text {N is the total number of images.} \end{aligned}$$

Mean Intersection over Union (IoU) computes the overlap of the ground truth and prediction and is defined as follows:

4 $$\begin{aligned} MIoU = \frac{1}{N} \sum _{i=1}^{N} \frac{TP}{TP + FP + FN}, \quad \text {N is the total number of images.} \end{aligned}$$

With the performance metric defined, the validation dataset is used to understand the model’s overall performance on untrained images. Even though the model computes the loss on the validation dataset in every epoch, it never “learns” from the validation loss. In other words, the weights of the model are frozen during validation loss computation. In addition to the validation dataset, the *test* dataset is also introduced to evaluate the trained model’s performance on novel data. Adding test data to further evaluate performance of trained model is of common practice^38–40^.

**Figure 5:**
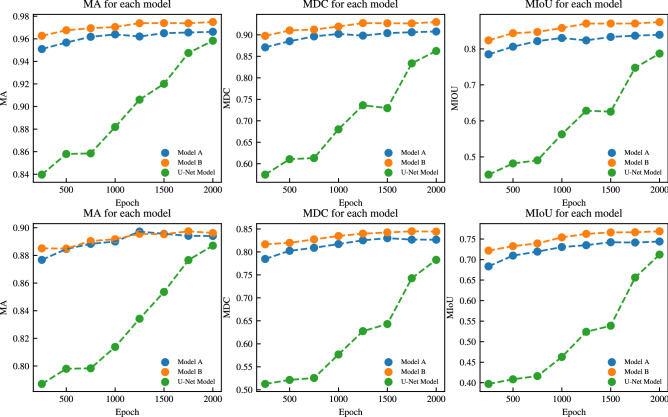
The performance metrics were computed for both the validation dataset (top row) and test dataset (bottom row) every 250 epochs up to 2000.

The performance metrics computed on both validation and test data are shown in Fig. 5. We can see that the model performance improves as the number of epochs increases. However, the performance with the test dataset decreases as compared to that with the validation dataset. This is expected as most of the model training contained data collected from a single setup shown in Fig. 1. One direction for future work would be to increase the training data size which captures different setups of spray in different environments, not just the lab setting that we have. Nonetheless, the performances of both proposed models are still better than that of the U-Net model.

## Inference

To this point, it has been clearly illustrated that both of the proposed models (*model A* and *model B*) have fewer training parameters, smaller model sizes, and take less time to train as compared to U-Net. Therefore, there is a compelling motivation to pick either of the proposed models (*model A* and *model B*) for further spray pattern recognition. *Model A* and *Model B* share a lot of similarities in terms of network architecture, model size, and training time. Compared to *model A*, *model B* has lower values of training and validation loss. Furthermore, *model B* performs better in terms of mean accuracy, mean dice coefficient, and mean intersection over union in comparison with *model A* in the validation dataset. Model B also performs better on the test dataset compared to Model A (see Fig. 5). This highlights the efficacy of Model B in accurately predicting the target outcomes and underscores its potential as a robust model for spray boundary detection.

**Figure 6:**
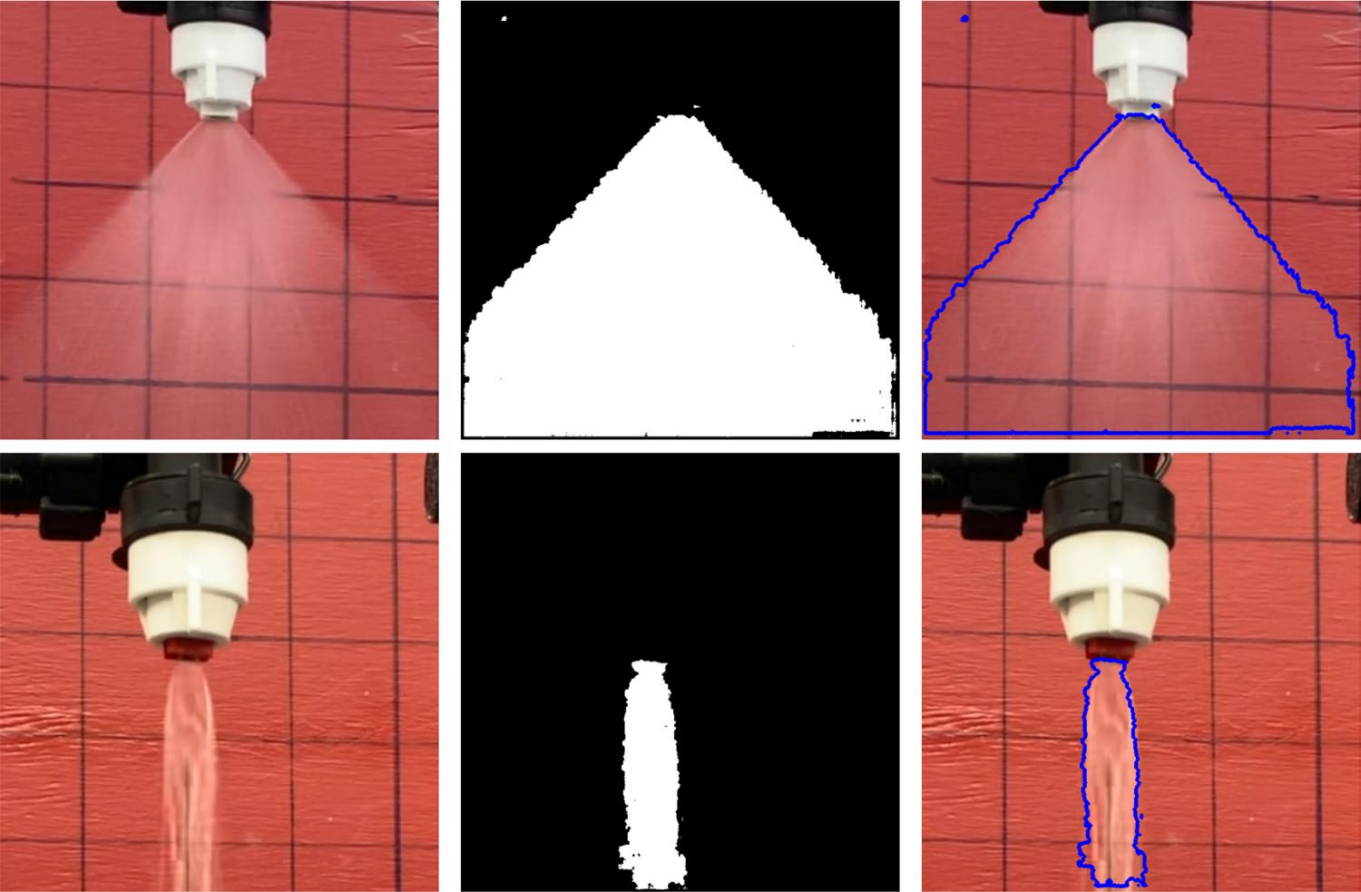
Input images to the left column, output binary mask obtained from *model B* to the middle column, and superimposing the boundary from the output mask to the input image to the right column.

Thus, *model B* was selected over *model A* for further analysis. The results also clearly show that $1\times 1$ convolution can be used efficiently within the model for the task of image segmentation. This is seen through their close values for the training loss $L_T$ and validation loss $L_V$ shown in Fig. 4. Figure 6 depicts the typical results of Model B in segmenting spray images to detect spray patterns. Even though the model can recognize the regions of spray in the given images, as with all deep-learning models, some loss remains when training. In our case, both training and validation losses are less than 0.1. With the ability to segment the region of spray in the given image, the next step would be to estimate the spray cone angle.

## Estimation of spray cone angle

This section discusses the post-processing carried out to fit a standard geometry to the detected spray pattern. The post-processing algorithm consists of the following steps:

**(i) Binary threshold:** One channel output image with a pixel value between 0 and 1 inclusive obtained from *Model B* is scaled to a gray-scale image with a pixel value between 0 and 255. This is because spray cone angle estimation uses the classical computer vision library *OpenCV*. *OpenCV* library functions expect the pixels to have a value between 0 and 255. Threshold is applied to the gray-scale image. The technique works by assigning each pixel either a value of 0 or 255 based on the following equation:

5 $$\begin{aligned} I_B = \sum _{i=1}^{W} \sum _{j=1}^{H} i_b[i,j] \quad {\left\{ \begin{array}{ll} 255 &{}\quad \text {if }i_b[i,j] \ge \text { threshold}\\ 0 &{}\quad \text {else} \end{array}\right. } \end{aligned}$$

where $i_b$ is the output image from applying the *model B* to a given input image *x*, *W* and *H* are width and height, respectively, of the image $i_b$. The *threshold* value can be chosen by the user with the value of 200 selected in our case. With the binary thresholding operation, regions of spray are represented by white color (pixel value of 255) and everything else is black (pixel value of 0).

**Figure 7:**
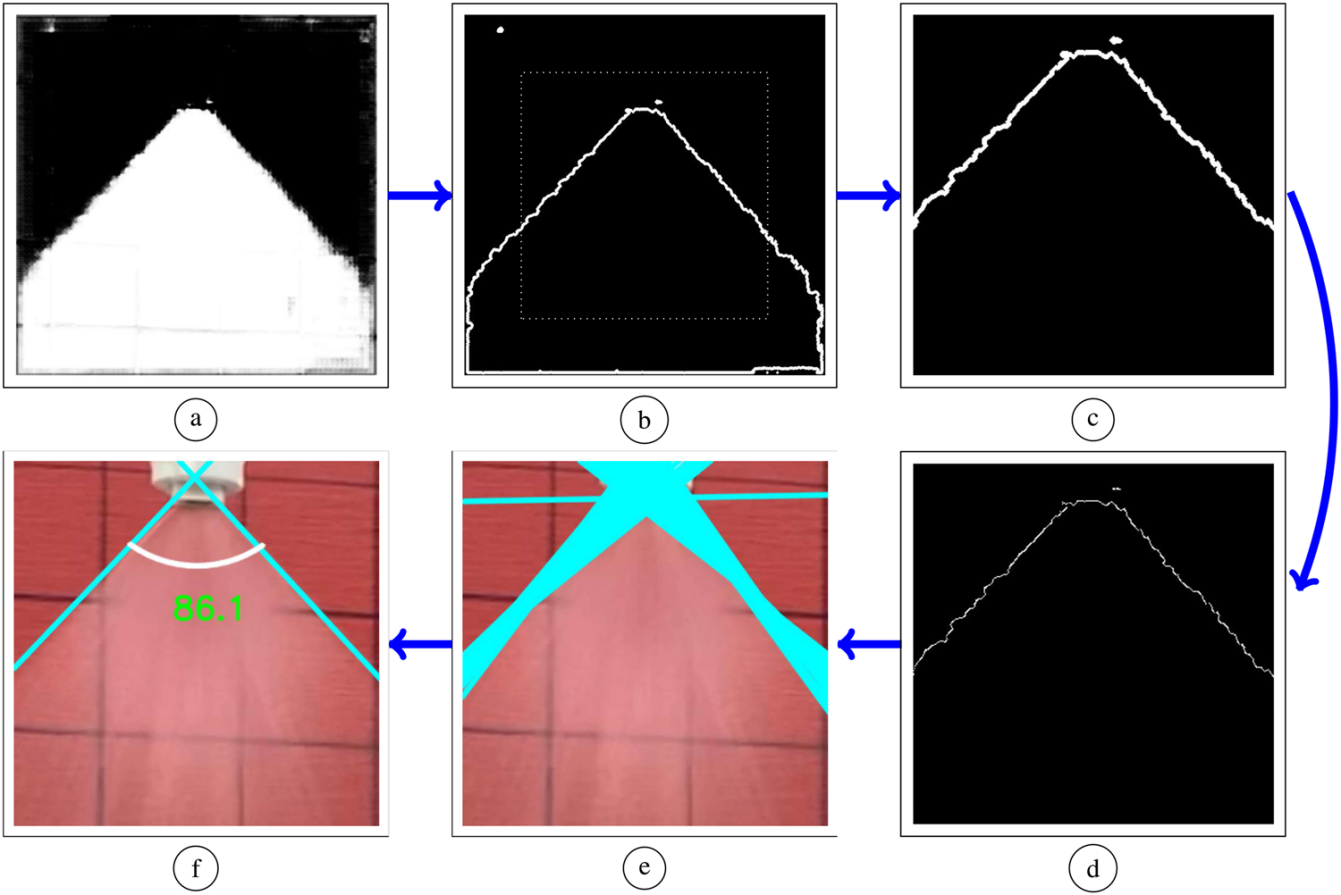
Post processing to estimate spray cone angle. (**a**) The output from the deep-neural network for segmentation. (**b**) The output of boundary detection (**c**) The output of cropping. (**d**) The output of image thinning. (**e**) The output of lines fitting. (**f**) The output of line refinement and angle computation.

**(ii) Spray boundary detection:** With the spray region detected next step is to detect the edge/boundary of the detected spray region. The spray boundary can be detected using an edge detection technique. For edge boundary extraction contour is extracted^41^. This technique enables us to establish the topological relationship between the spray boundary and its surroundings. This topological structure can be helpful to remove any false detection spots inside the sprayer pattern. The output of this step is shown in Fig. 7b.

**(iii) Cropping:** The next step would be to focus on the nozzle region, where liquid exits the nozzle. To do so, the image obtained after applying the above step is cropped from the center to contain $80\%$ of the total pixels. In particular, $20\%$ of the pixels surrounding the image boundary is removed resulting in an image size of $342\times 342$. This cropped image is referred to as $I_c$. The output of this step is shown in Fig. 7c and the cropping window is seen in Fig. 7b as the dotted rectangle.

**(iv) Image thinning:** In this module, redundant pixel values representing the edge of the spray are removed via an image thinning process. The main idea behind image thinning is to reduce foreground regions to a remnant such that original connectivity is preserved. Specifically, this operation removes redundant white pixels, where white pixels represent the edge of the spray. The thinning algorithm^42^ is represented using the below equation:

6 $$\begin{aligned} I_e = I_e \vee (I_c - f_1(I_c)) \quad \text {and} \quad I_c = f_2(I_c) \end{aligned}$$

where $f_1$ is the erosion followed by dilation and $f_2$ is the erosion operation. In both morphological operations, a $3\times 3$ structural element is used. Operation $\vee$ is the bit-wise “OR” operation. Initially, $I_e$ is a one-channel image with all pixel values of zero and $I_c$ is the initially cropped image. Both images $I_e$ and $I_c$ are repeatedly updated and the operation as defined in eqaution (6) is repeated unless all pixel values in $I_c$ are zeros. The output of this step is shown in Fig. 7d.

**Figure 8:**
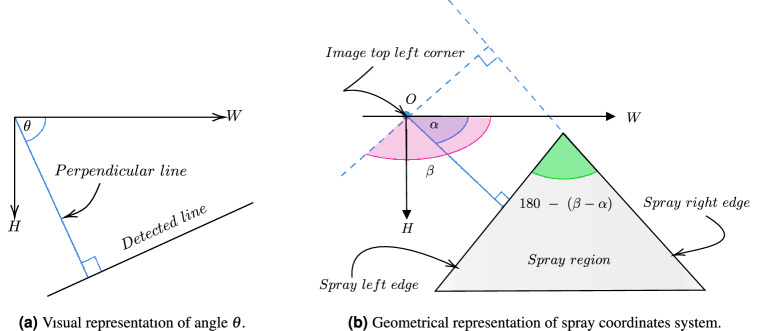
Spray cone angle estimation using computer vision.

**(v) Lines fitting:** The output from the above step provides rudimentary pixels representing the boundary of a spray pattern. Using the Hough line transformation technique^43^, lines represented in polar coordinates are fitted to the above output image. The condition for a line to fit into the image requires that there are least 20 continuous boundary pixels to form a line. The value of 20 was chosen to detect as many lines as possible. Based on our observation, there is no significant impact on choosing different values in the range of 20 to 130. Out of all the fitted lines, those that represent the left side of the spray boundary and the right side of the spray boundary are filtered outs. This is because the left and right edges defined the spray cone angle. To do so, the following logic is applied over all detected lines, and those lines that satisfy the below logic are selected:

7 $$\begin{aligned} \text {accept a detected line} {\left\{ \begin{array}{ll} &\quad \text {if } \theta \ge 20^\circ \text { and } \theta \le 85^\circ \\ &\quad \text {or } \theta \ge 95^\circ \text { and } \theta \le 160^\circ \end{array}\right. } \end{aligned}$$

where $\theta$ is the angle between the image width direction and a perpendicular line to the detected line as depicted in Fig. 8a. All accepted lines are grouped into two sets $Q_1$ and $Q_2$ where $Q_1$ contains lines slope that have $\theta \ge 20^\circ$ and $\theta \le 85^\circ$; and set $Q_2$ contains lines with $\theta \ge 95^\circ$ and $\theta \le 160^\circ$.

**(vi) Line refinement and angle computation:** A spray cone angle can be obtained by computing the angle between the left boundary and the right boundary. From the previous steps, there could be multiple lines in set $Q_1$ and $Q_2$. Let $\alpha$ be the average of all the slope values stored in set $Q_1$ and $\beta$ be the average of all the slope values stored in the set $Q_2$. Hence, $\alpha$ and $\beta$ lead to two lines, one of which represents the left boundary and the other represents the right boundary of the spray region. Refer to Fig. 8b for a visual representation. Finally, the spray cone angle in degrees can be computed as $180 - (\beta - \alpha )$.

## Validation

Given a spray image, *model B* can be used to compute the binary spray pattern. Using the approach above, the spray cone angle can be estimated for each binary spray pattern image. The spray cone angle computed from the above step is compared with the manual measurements. To obtain these manual measurements, the same images that are processed by *model B* are loaded into ImageJ^44^. This is the image processing software developed by the National Institutes of Health and the Laboratory for Optical and Computational Instrumentation. Image J has been widely used for the validation of spray angles for different applications^9,45–47^. Spray angles obtained using ImageJ are referred to as manual measurements (the current state of the art) while the results obtained by our proposed method are referred to as estimated values (as illustrated in Fig. 7). Ten images are randomly selected and the results are shown in Table 5.

**Table 5:** Validation and verification. All units are in degrees.

Manual measurements	Estimated angles	Errors	Relative errors $\%$
86.4	86.1	0.3	0.347
89.3	89.9	0.6	0.672
90	91.8	1.8	2.000
88	90.9	2.9	3.295
68.98	70.5	1.52	2.204
86.2	87.1	0.9	1.044
80.98	79.4	1.58	1.951
79.01	80.1	1.09	1.380
58.2	57.7	0.5	0.859

As seen in Table 5, our proposed method’s results align with the manual measurements within the relative error of less than $3.3\%$. This validation step further reassures the effectiveness of the proposed method. Thus, the pipeline developed can be used to estimate the spray cone angle in place of manual measurements using ImageJ.

## Conclusion

This work lays the foundation for deep-learning techniques to better understand the sprayer systems’ characteristics, as well as to address other problems in agriculture. It demonstrates that $1\times 1$ convolution can play a significant role in the detection of spray patterns which leads to a model with fewer parameters and higher efficiency. The trained model also demonstrates the excellent capability of segmenting spray regions. Because the output from the trained model is used to estimate the spray cone angle, the data analysis is robust to the lighting condition. This also removes the need for using a special camera to estimate the spray cone angle. An additional benefit of using a deep learning approach is that the model can be trained with additional data such as images of different resolutions, different outdoor settings, and images taken from different cameras to make the model more general and reliable.

Though this work does not go into the detail of the actual implementation of the overall algorithm in the system out in the field, the application is always a motivational factor. Successful field tests will require processing capability at a high frame rate since the environment is substantially more dynamic as compared to the controlled environment in the lab. In the current paper, all the codes were written in Python. The post-processing tasks and the trained model were run using an old computer with a 2.9 GHz Dual-Core Intel Core i5 GPU, 8 GB 1867 MHz DDR3 memory, and Intel Iris Graphics 6100 card. The device was able to segment the region in the spray and also estimate the spray cone angle at the rate of 3 frames per second. For future field tests, coding in a lower-level programming language like C/C++ and with a more powerful computer and optimized code should lead to a higher processing frame rate. In future research, we will develop deep-learning algorithms for segmenting spray patterns under the dynamic influence of wind. We also plan to validate the proposed algorithms in field tests.

## Data Availability

The data that support the findings of this study are available from the corresponding author upon reasonable request within regulations for data protection.
